# Supplementary material for: Exploiting the aggregation propensity of beta-lactamases to design inhibitors that induce enzyme misfolding
Source: Nat Commun. 2023 Sep 9;14:5571. doi: 10.1038/s41467-023-41191-z (PMC10492782; doi:10.1038/s41467-023-41191-z)
Supplement: Supplementary file 1 — Supplementary Information [file 41467_2023_41191_MOESM1_ESM.pdf]

SUPPLEMENTARY DATA

Exploiting the structural vulnerability of beta-lactamases to design inhibitors that induce enzyme misfolding

Ladan Khodaparast[1,2,#], Laleh Khodaparast[1,2,#], Guiqin Wu[1,2], Emiel Michiels[1,2], Rodrigo Gallardo[1,2], Bert Houben[1,2], Teresa Garcia[1,2], Matthias De Vleeschouwer[1,2], Meine Ramakers[1,2], Meine Ramakers[1,2], Hannah Wilkinson[1,2], Ramon Duran-Romaña[1,2], Johan Van Eldere[3], Joost Schymkowitz[1,2,*] and Frederic Rousseau[1,2,*]

Supplementary Figures

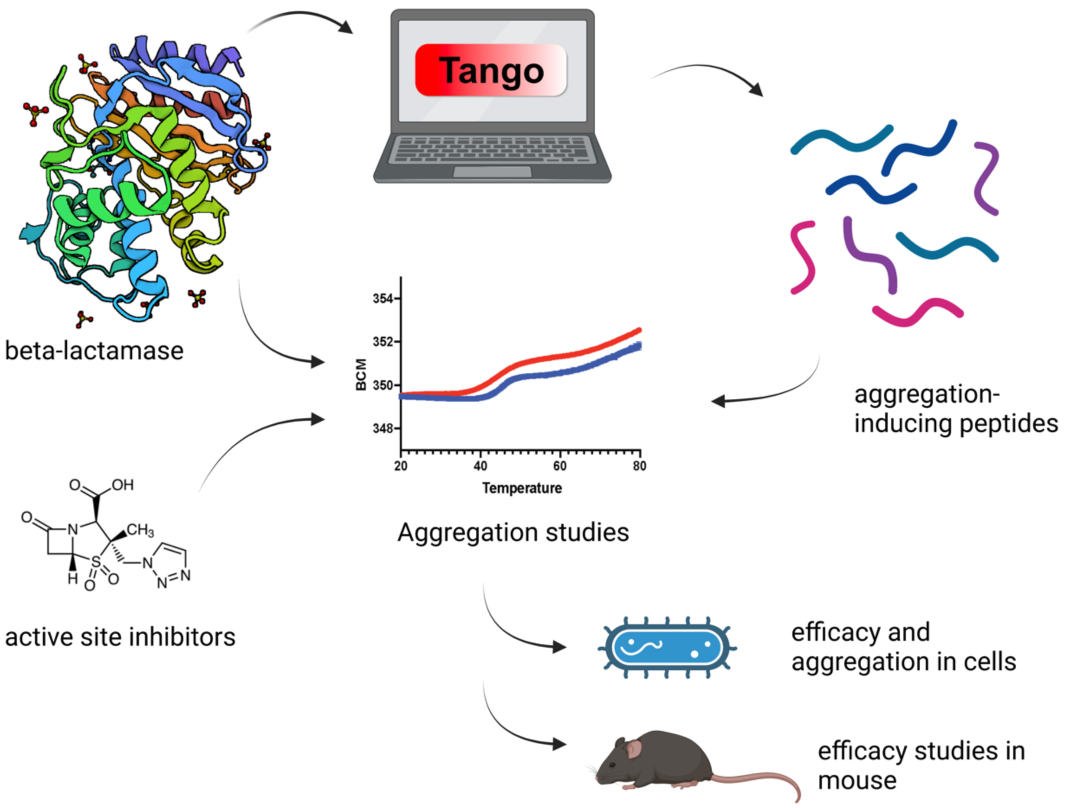

Supplementary Figure 1: Schematic overview of the work presented in this manuscript. Created with BioRender.com

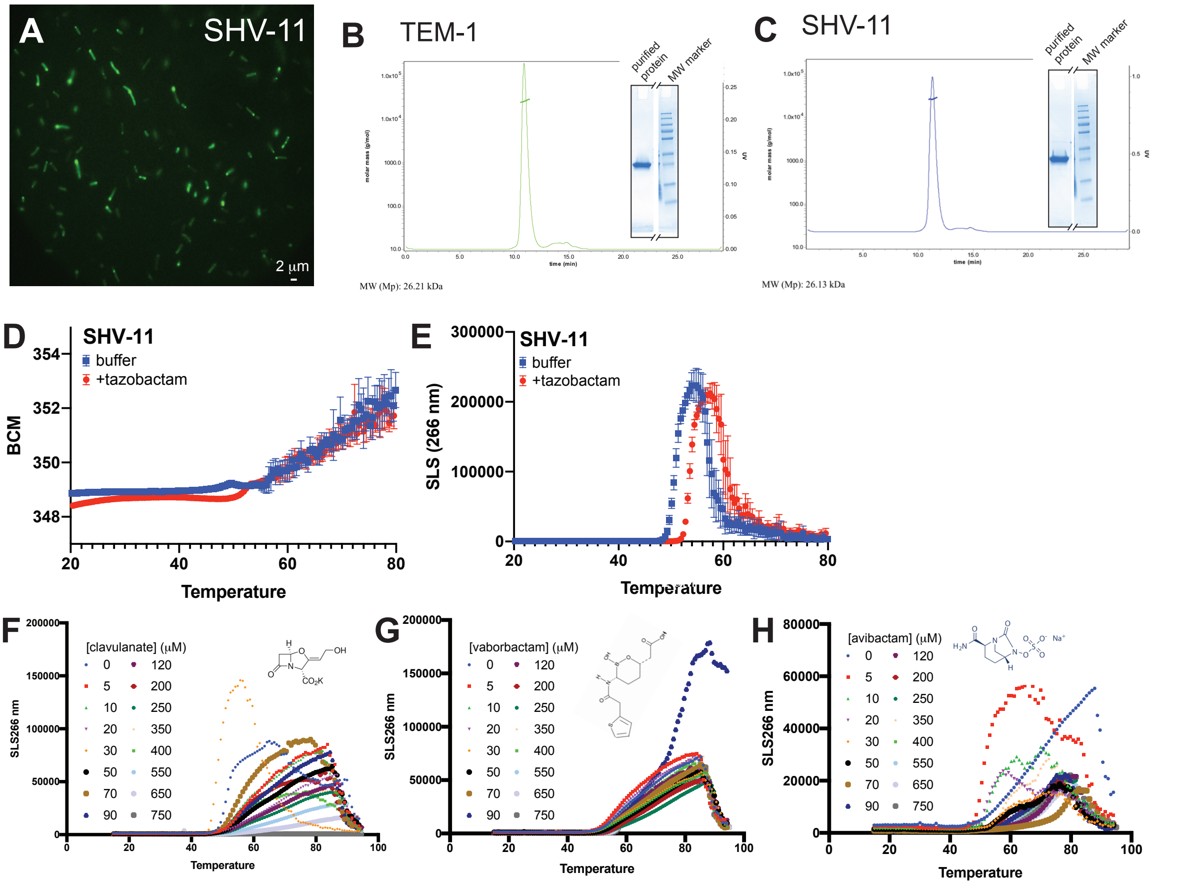

Supplementary Figure 2: (A) Structured illumination Microscopy (SIM) super-resolution image of *E. coli* BL21 overexpressing a GFP-fusion of SHV-1. A single representative micrograph is shown of one 3 independent repeats performed. (B) Elution profile of recombinantly purified TEM-1 upon size-exclusion chromatography followed by Multi-Angle Light Scattering mass detection (SEC-MALS). The continuous green line is the UV signal (right Y-axis), the dots near the center of the peak are the molecular masses measured in parallel (left Y-axis). Inset: Coomassie-stained SDS-PAGE of the same sample. The data show single representative run of one of 3 independent repeats. (C) Elution profile of recombinantly purified SHV-11 upon size-exclusion chromatography followed by Multi-Angle Light Scattering mass detection (SEC-MALS). The continuous green line is the UV signal (right Y-axis), the dots near the center of the peak are the molecular masses measured in parallel (left Y-axis). Inset: Coomassie-stained SDS-PAGE of the same sample. The data show single representative run of one of 3 independent repeats. (D) Heat denaturation of SHV-11 monitored by intrinsic fluorescence plotted as the Bary Centric Mean (BCM) of the emission spectrum in the presence (red) and absence (blue) of tazobactam. The melting temperature (Tm) is derived from these data. The plot shows the mean of 7 replicates, and the error bars represent the standard deviation. (E) Temperature-dependent evolution of the Right-Angle Light Scattering (RALS) intensity measured simultaneously with the data in C to monitor protein aggregation. The aggregation onset temperature Tagg is derived from these data. The plot shows the mean of 7 replicates, and the error bars represent the standard deviation. (F, G, H) Temperature-

dependent evolution of the Right-Angle Light Scattering (RALS) intensity as a function of beta-lactam inhibitors: clavulanate (H), vaborbactam (I) and avibactam (J). Each curve a single experiment, which was repeated in triplicate.

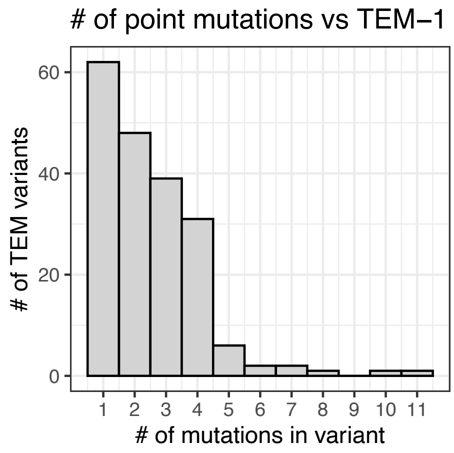

Supplementary Figure 3: Histogram of the number of point mutations that separate the TEM variant described in the Beta-Lactamase DataBase (BLDB) from the wild-type protein TEM-1.

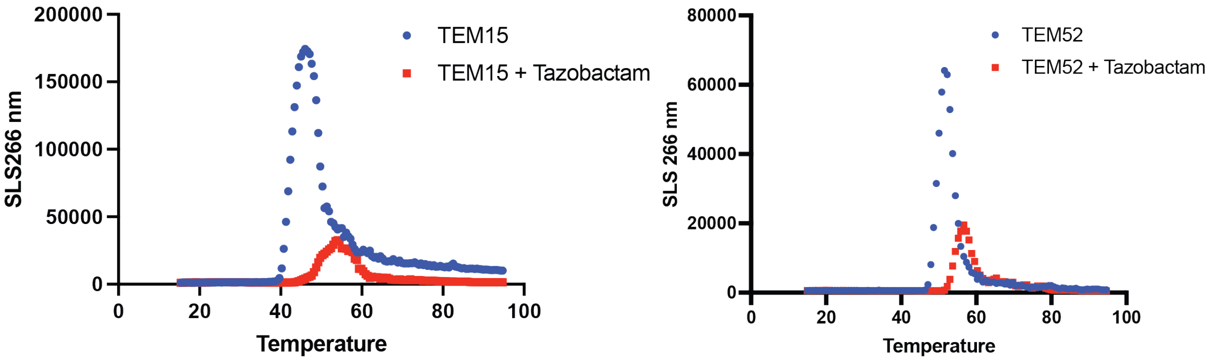

Supplementary Figure 4: Aggregation of TEM15 and TEM52 during a temperature ramp, in the presence and absence of tazobactam, measured by Static Light Scattering. A single replicate is shown.

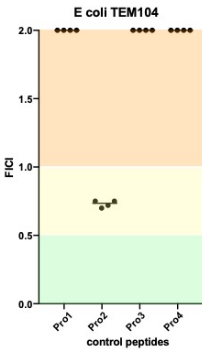

Supplementary Figure 5: Fraction Inhibitory Concentration Index (FICI) values obtained from checkerboard assays between control peptides 1-4 and penicillin G. The peptide sequences were obtained by introducing proline mutations in TEM3.2 as follows: Pro1

(*RPTAPLHNRRPRPTAPLRQRR*), Pro2 (*RLPPFLHNRRPRLPPFLRQRR*), Pro3 (*RPTPFLHNRRPRPTPFLRQRR*) and Pro4 (*RLTPFPHNRRPRLTPFPRQRR*). Each of the five dots per condition indicates an independent experiment of the measurement consisting of 96 datapoints. FICI values below 0.5 indicate synergy (shaded in green). Values between 0.5 and 1.0 indicate additivity (shaded in yellow) and values greater than 1 indicate indifference (shaded in red) between the combined substances.

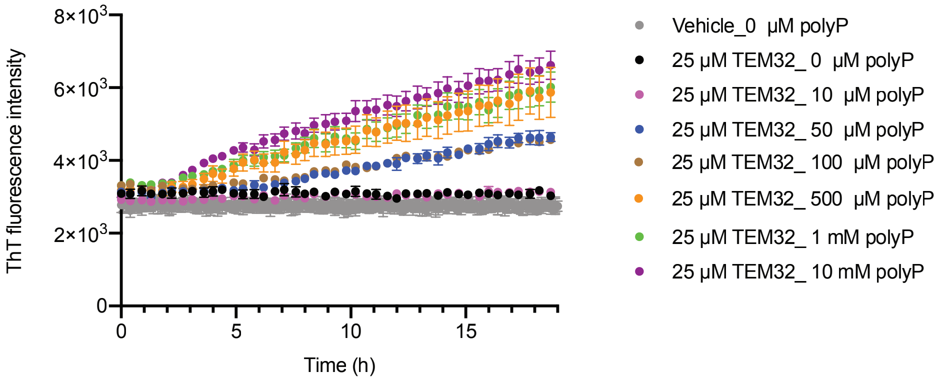

Supplementary Figure 6: Dose-dependent effect of poly-P on the aggregation of peptide TEM3.2. The data shown are the mean and SEM of 9 technical replicates (for aggregation assays it is important to show the level of variability between technical replicates). The experiment was done in 3 biological repeats.

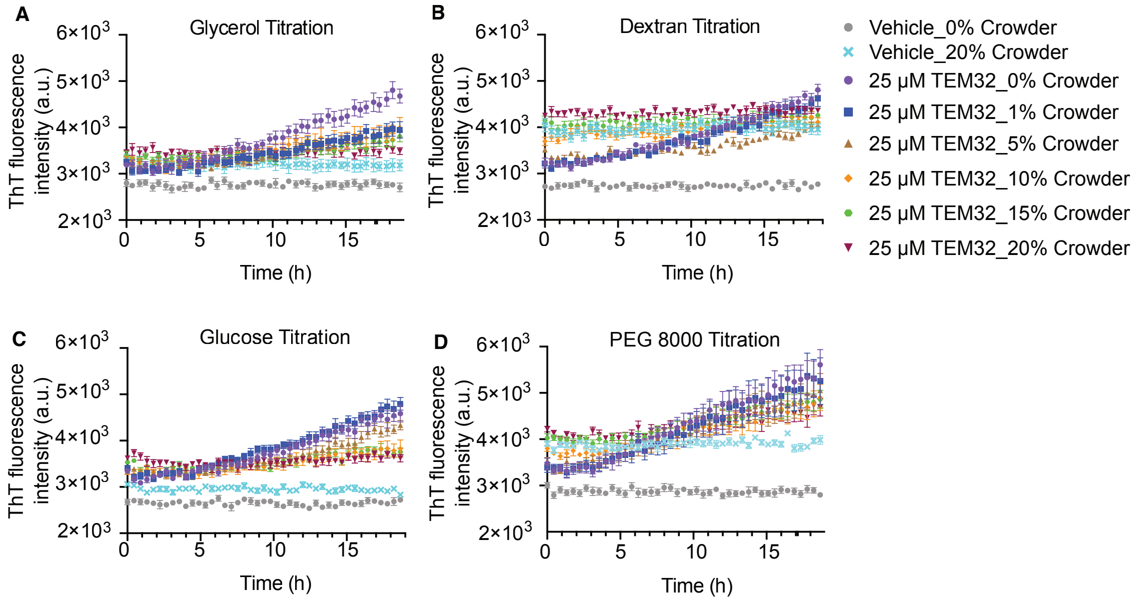

Supplementary Figure 7: Effect of molecular crowding on the aggregation in vitro of the TEM3.2 peptide. The panels describe the effect of (A) Glycerol, (B) Dextran, (C) Glucose and (D) PEG 8000. The data shown are the mean and SEM of 9 technical replicates (for aggregation assays it is important to show the level of variability between technical replicates). The experiment was done in 3 biological repeats.

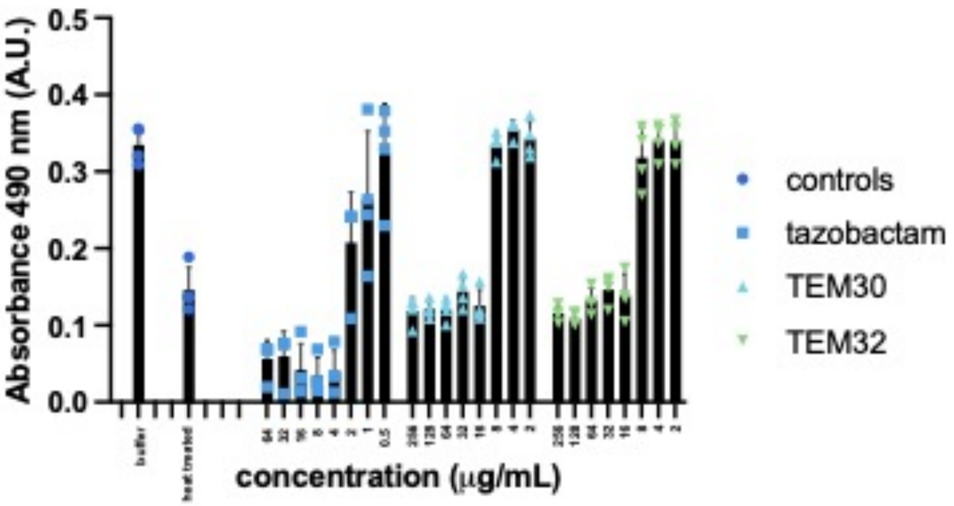

Supplementary Figure 8: Conversion of the chromogenic beta-lactamase substrate nitrocefin of recombinantly purified TEM-1 treated for 1 hour at 37 C with the indicated concentration of tazobactam or Pept-In, as well as heat-denatured control condition. The individual measurements are shown as dot (4 repeats), the bars indicate the mean and the error bars the standard deviation.

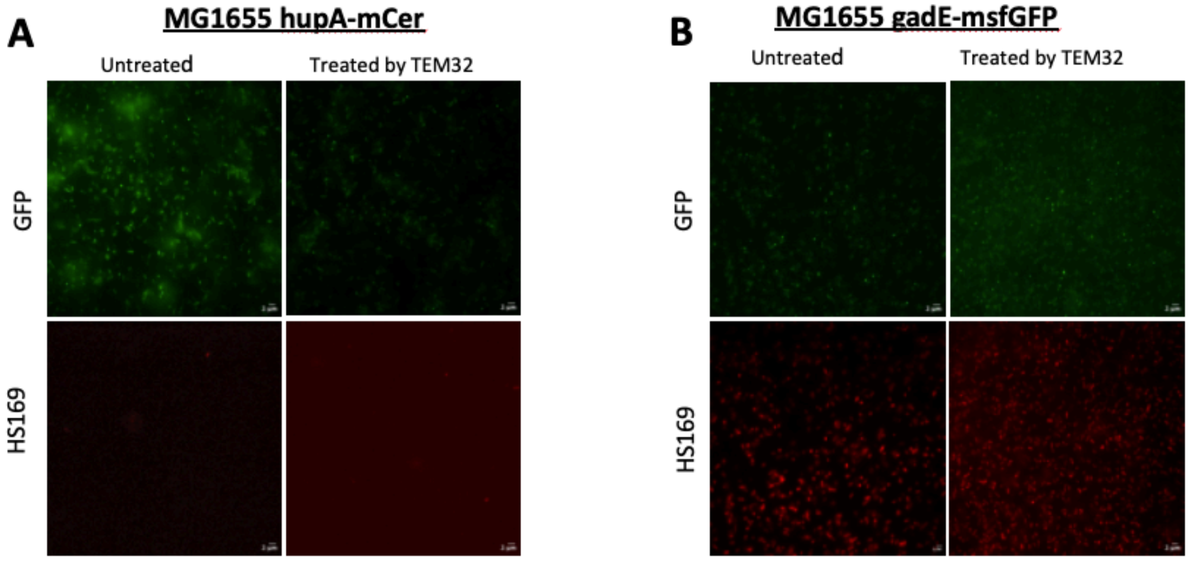

Supplementary Figure 9: Effect of peptide TEM3.2 on the subcellular localization of fluorescent protein fusions in *E. coli*. (A) Fusion between hupA (*E. coli*) and the cerulean FP. (B) Fusion between gadE (*E. coli*) and the super-folder GFP.

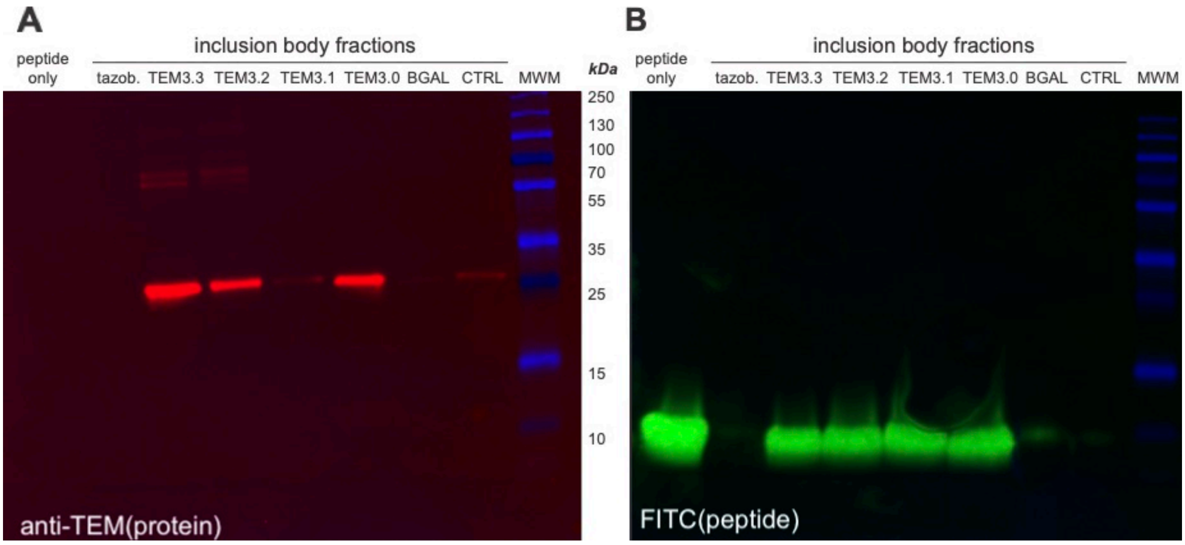

Supplementary Figure 10: (A) Full western blot of the gel of the inclusion body fraction of *E coli* TEM-1 treated with the FITC-labelled peptides as indicated and controls. The green fluorescence corresponds to the anti-TEM antibody. The boxed segment is analyzed by mass spectrometry in Suppl Figure 7. (B) FITC signal of the labelled peptides.

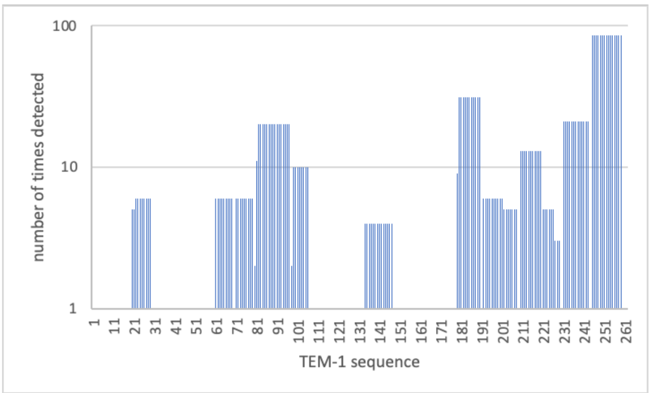

Supplementary Figure 11: Mass spectrometric analysis of a tryptic digest of the lane corresponding to the TEM protein from supplementary Figure 6, showing the peptide coverage over the sequence, confirming that the protein is being detected by the antibody is indeed the TEM beta-lactamase.

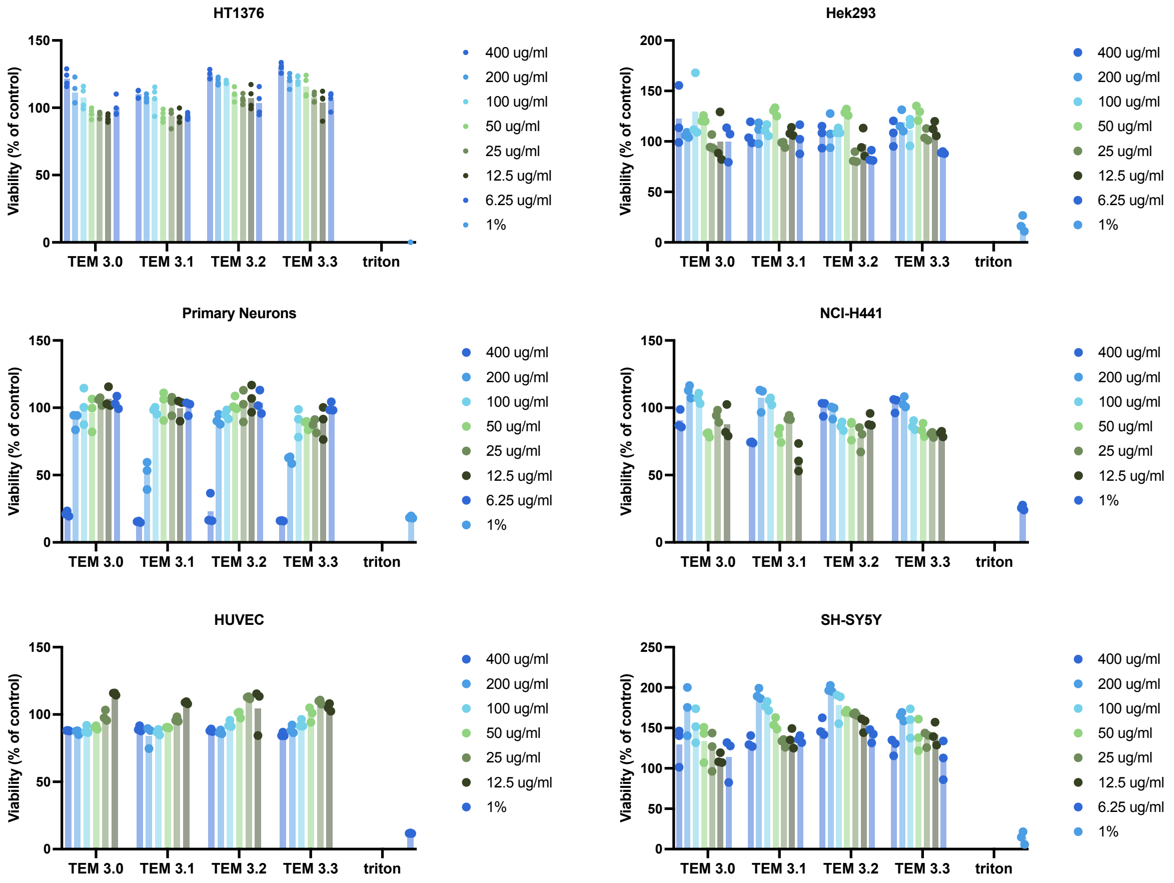

Supplementary Figure 12: Cell viability assays on mammalian cells using the CellTiter Blue assay, plotted as relative values compared to vehicle. The colours of the bars show the concentrations indicated in the legends in µg/mL, ranging from 400 to 6.25 µg/mL. The dots indicate individual measurements on independent wells of cells (4 repeats), the bars indicate the mean.

A

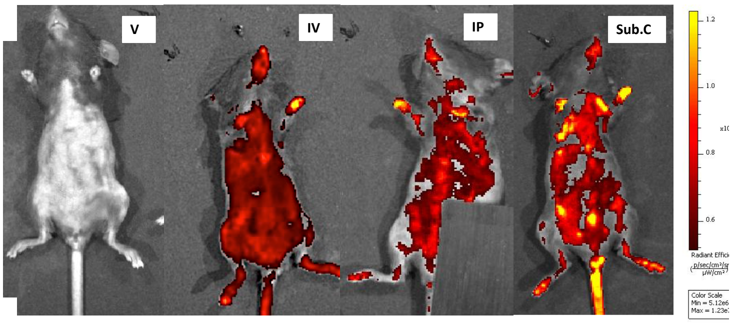

B

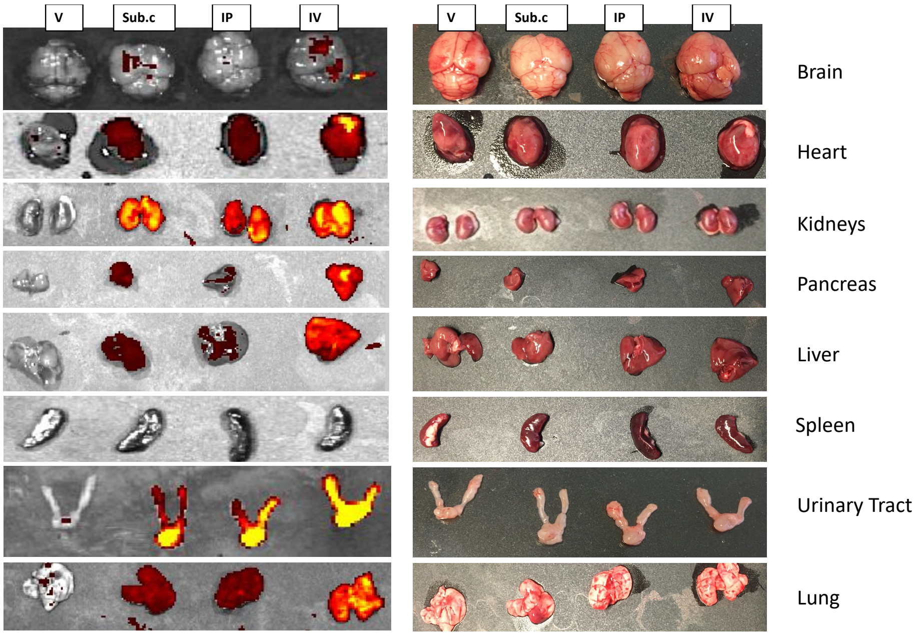

Supplementary Figure 13: *In vivo* fluorescence images of mouse at 1h post-injection FITC labeled TEM3.2. (A) At ventral position; 1h post injection of peptides (10 mg kg-1), Intravenous (IV), Intraperitoneal (IP); side of injection has covered since the high intensity of the side of injection interfere imaging, subcutaneous (Sub.c) and vehicle (V) treated. (B) *Ex vivo* Fluorescence imaging of organs injected with peptides 1h post-injection ((10 mg kg-1) with a different way of administration (left panel), the organs images (right panel) shows the peptides reaches the target organs.), Intravenous (IV), Intraperitoneal (IP, subcutaneous (Sub.c) and vehicle (V). Images was taken by *in vivo* optical imaging system, IVIS® Spectrum system, the Laser scanning employed to determine FITC-labeled peptides.

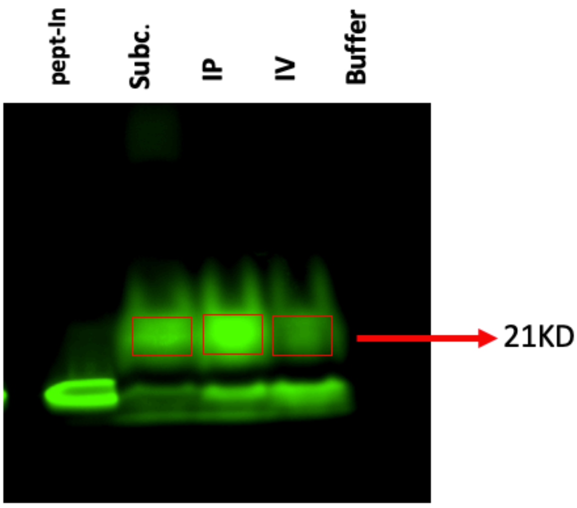

Supplementary Figure 14: SDS-PAGE analysis of FITC-labelled TEM3.2 peptide taken from the bladder of animals 1h after sub-cutaneous, Intraperitoneal, and Intravenous administration. Intact peptide as well as peptide bound to a mouse protein can be detected. The protein was identified by mass spectrometry following tryptic digest to be Major Urinary Protein 1 (MUP1).

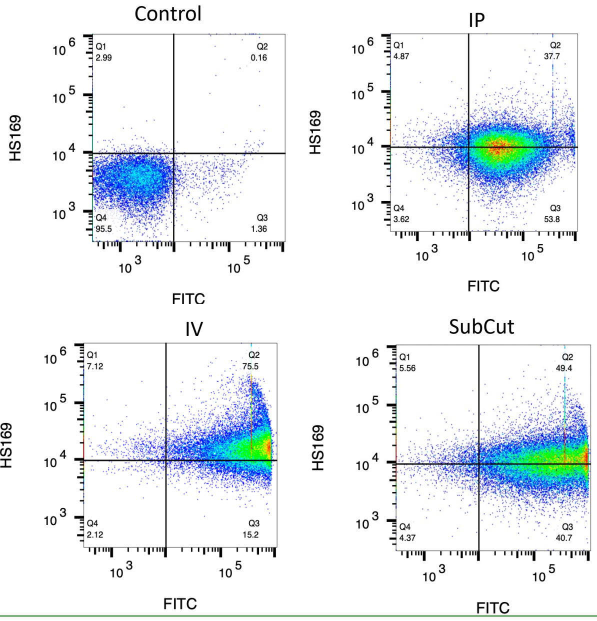

Supplementary Figure 15: FACS analysis of uro-pathogenic *E. coli* strain UPEC_blaTEM-1 taken from the bladder of infected mice, 2h hours after treating with TEM3.2 (IP; Intraperitoneal, IV; intravenous, SubCut; subcutaneous injections) showing peptide uptake on the X-axis and protein aggregation (red out as fluorescence of the HS169 compound) on the Y-axis.

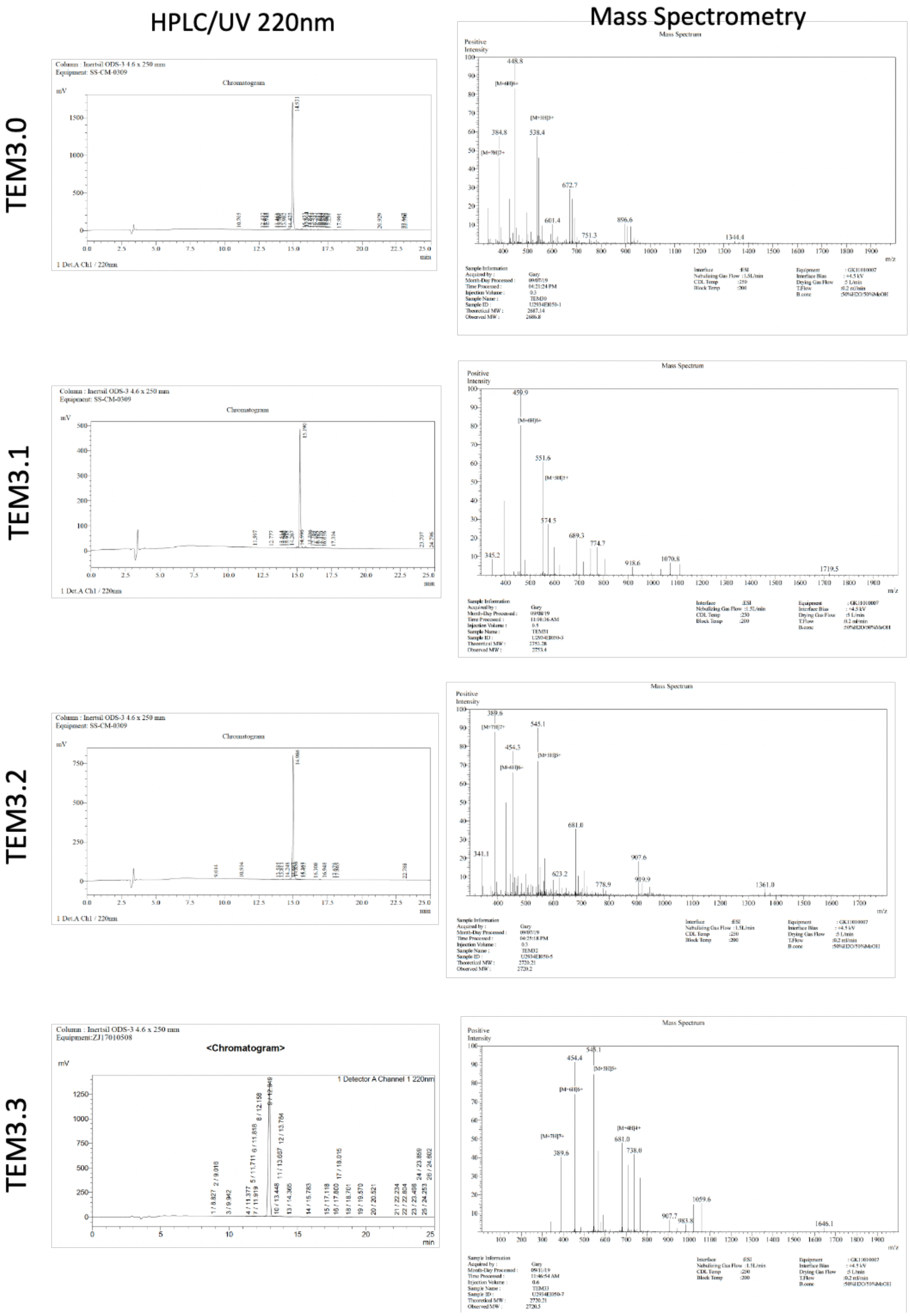

Supplementary Figure 16: Peptide quality control data from HPLC and Mass Spectrometry.

Supplementary Tables

Supplementary Table 1: Kinetic parameters for different beta-lactamases against Amp.

	Km (µM)	Kcat (s-1)	Ref
**TEM-1**	50	1428	[1]
**NDM-5**	117.5	1912	[2]
**OXA-48**	395	955	[3]
**VIM-1**	917	37	[4]

Supplementary Table 2: The antibiotic resistance profiles of bacterial strains used in this study. VITEK® 2 AST Cards used for antimicrobial susceptibility testing. Abbreviations: S, susceptible; I, intermediate; R, resistant.

Antimicrobial agent	*E. coli* TEM-104		*E. coli* TEM-206		*E. coli* TEM-30		*E. coli* TEM-148		*E. coli* TEM-16		*E. coli* NDM5a		*E. coli* NDM5b		*E. coli* SHV1 (APHA)	
	MIC	Interoretation	MIC	Interoretation	MIC	Interoretation	MIC	Interoretation	MIC	Interoretation	MIC	Interoretation	MIC	Interoretation	MIC	Interoretation
Ampicillin	>=32	R	>=32	R	>=32	R	>=32	R	>=32	R	>=32	R	>=32	R	>=32	R
Amoxicillin + clavulanic acid	>=32	R	4	*R	16	*R	4	*R	16	*R	>=32	R	>=32	R	16	R
Piperacillin / tazobactam	16	*I	<=4	*R	<=4	*R	<=4	*R	<=4	*R	>=128	R	>=128	R	>=128	R
Temocillin	8	S	16	S	<=4	S	<=4	S	>=32	R	>=32	R	>=32	R	<=4	R
Cefuroxime	4	S	>=64	R	>=64	R	>=64	R	>=64	R	>=64	R	>=64	R	>=64	R
Cefuroxime Axetil	4	S	>=64	R	>=64	R	>=64	R	>=64	R	>=64	R	>=64	R	>=64	R
Cefotaxime	<=1	S	>=64	R	4	R	16	R	>=64	R	>=64	R	>=64	R	>=64	R
Ceftazidime	<=1	S	16	R	<=1	*I	<=1	*I	16	R	>=64	R	>=64	R	>=64	R
Cefixime	<=1	S	>=64	R	<=1	*I	2	*I	>=64	R	>=64	R	>=64	R	16	R
Cefoxitin	<=4	S	<=4	S	8	S	<=4	S	8	S	>32	R	>32	R	>32	R
Meropenem	<=0.25	S	<=0.25	S	<=0.25	S	<=0.25	S	<=0.25	S	>=16	R	8	I	<=0.25	S
Levofloxacin	<0.12	S	>=8*	R	>=8*	R	1*	S	<=0.12	S	>=8	R	>=8	R	<=0.12	S
Nitrofurantoin	<=16	S	<=16	S	32	S	32	S	<=16	S	64	R	64	R	<=16	S
Gentamicin	<=1	S	<=1	S	>=16	R	<=1	S	<=1	S	>=16	R	<=1	S	>=16	R
Tobramycin	<=1	S	<=1	S	8	*R	<=1	S	<=1	S	8	R	<=1	S	4	*R
Amikacin	<=2	S	<=2	S	<=2	S	<=2	S	<=2	S	<=2	S	<=2	S	<=2	S
Trimethoprim/sulfamethoxazole	<=20	S	>=320	R	40	S	>=320	R	<=20	S	<=20	S	>=320	R	>=320	R
Tigecycline	<=0.5	S	<=0.5	S	2	I	<=0.5	S	<0.5	S	<0.5	S	<=0.5	S	<=0.5	S
Colistin	<=05	S	<=0.5	S	<=0.5	S	<=0.5	S	<=0.5	S	<=0.5	S	<=0.5	S	<=0.5	S

Supplementary Table 3: Fractional inhibitory concentration index (FICI) values calculated for the tested strains/peptides.

FIC index ≤ 0.5, the combination of antibiotics has a synergistic effect,
FIC index of 0.5–1.0 indicates that the effects of the drugs are additive,
FIC is between 1 and 4, the combination effect is indifferent,
FIC ≥ 4 indicate an antagonistic combination.

E.coli TEM104	TEM 3.0 Peptide	TEM 3.1 Peptide	TEM 3.2 Peptide	TEM 3.3 Peptide	BGAL Peptide
Replicate1	0,254	0,75	0,188	0,133	1
Replicate2	0,25	0,625	0,141	0,129	1
Replicate3	0,375	0,5	0,188	0,375	1
Replicate4	0,127	0,5	0,313	0,313	1
Replicate5	0,25	1	0,127	0,133	-

E.coli SHV11	TEM 3.0 Peptide	TEM 3.1 Peptide	TEM 3.2 Peptide	TEM 3.3 Peptide	BGAL Peptide
Replicate1	0,75	0,25	0,156	0,375	2
Replicate2	0,75	0,375	0,25	0,25	1
Replicate3	0,5	0,281	0,281	0,188	1
Replicate4	0,75	0,375	0,375	0,281	1
Replicate5	0,75	0,126	0,375	0,375	-

E.coli Kanamycin resistant	TEM 3.0 Peptide	TEM 3.1 Peptide	TEM 3.2 Peptide	TEM 3.3 Peptide	BGAL Peptide
Replicate1	0,75	0,75	2	2	2
Replicate2	1	1	2	2	2
Replicate3	1	1	2	2	2
Replicate4	1	1	2	2	2

TEM3.2 peptide	*E.coli* TEM 104	*E.coli* SHV11	*E.coli* TEM 30	E.coli TEM 206	*E.coli* TEM 148	*E.coli* TEM 163
Replicate1	0,188	0,156	0,094	0,25	0,375	0,094
Replicate2	0,141	0,25	0,07	0,5	0,25	0,125
Replicate3	0,188	0,281	0,094	0,375	0,25	0,156
Replicate4	0,313	0,375	0,066	-	-	0,039
Replicate5	0,127	0,375	0,375	-	-	-

NDM1-1 peptide	*E. coli* NDM-5a	E. coli NDM-5b	TEM104
Replicate1	0,37	0,156	0,5
Replicate2	0,38	0,214	0,78
Replicate3	0,21	0,34	0,72
Replicate4	0,195	0,25	0,05
Replicate5	0,25	0,16	0,75

NDM1-2 peptide	*E. coli* NDM-5a	E. coli NDM-5b	TEM104
Replicate1	0,375	0,156	0,75
Replicate2	0,36	0,25	1
Replicate3	0,25	0,29	0,5
Replicate4	0,159	0,31	1
Replicate5	0,25	0,181	0,72

BGAL peptide	*E. coli* NDM-5a	E. coli NDM-5b	TEM104
Replicate1	2	2	1
Replicate2	2	1	1
Replicate3	2	2	1
Replicate4	1,5	2	1
Replicate5	2	1,5	1,5

P33 peptide	*E. coli* NDM-5a	E. coli NDM-5b	TEM104
Replicate1	2	0,75	1
Replicate2	2	1	0,625
Replicate3	1,5	1	0,258
Replicate4	2	0,75	1
Replicate5	2	1,5	1

Supplementary Table 4: *In vivo* dose-escallation study with FITC-TEM3.2

Mice	Tox beheviour IV	Tox beheviour IP	Tox beheviour Sub
2 mg/Kg	no sign of tox	no sign of tox	no sign of tox
5 mg/Kg	no sign of tox	no sign of tox	no sign of tox
10 mg/Kg	no sign of tox	no sign of tox	no sign of tox
15 mg/Kg	no sign of tox	no sign of tox	no sign of tox
20 mg/Kg	no sign of tox	no sign of tox	no sign of tox

Supplementary Table 5: Hematological analysis of blood from animals treated for 14 consecutive days with FITC-TEM3.2.

	Parameter	Unit	Untreated		Treated		t-test
			AVRG	STDV	AVRG	STDV	(2 tails, 2 sample, unequal variance)
WBC	White blood cells		0,87	0,03	0,79	0,10	0,255
RBC	Red blood cells		2,00	0,10	1,93	0,15	0,513
HGB	Hemoglobin	g/dL	2,83	0,06	2,80	0,10	0,643
HCT	Hematocrit		9,80	0,50	9,60	0,56	0,667
MCV	Mean corpuscular vo	pg	50,20	3,15	35,03	25,49	0,364
MCH	Mean hemoglobin co	pg	14,33	0,40	14,80	1,08	0,523
MCHC	Mean hemoglobin co	g/dL	28,80	1,51	29,37	2,63	0,763
CHCM	mean optical hemogl	g/dL	29,13	1,53	27,83	0,12	0,216
CH	mean optical hemogl	pg	14,57	0,15	14,00	0,26	0,033
RDW	Red cell distribution width		15,63	0,78	12,97	0,21	0,005
HDW	Hemoglobin distribution width		2,03	0,08	1,78	0,01	0,006
PLT	platelets		214,33	65,31	204,67	29,14	0,826
MPV	Mean platelet volume		7,13	0,45	7,10	0,26	0,917
PDW	Platelet distribution width		49,63	7,92	42,50	0,53	0,195
PCT	Procalcitonin		0,15	0,06	0,15	0,03	0,858
MPC	mean platelet component		21,40	1,06	21,10	0,35	0,665
MPM	malignant pleural mesothelioma		1,37	0,02	1,34	0,03	0,224
LargePlt	LargePlt		1,00	0,00	0,33	0,58	0,116
Neut	Neutrophils		4,57	0,40	3,10	1,10	0,096
Lymph	Lymphocytes		89,17	0,23	91,50	0,75	0,007
Mono	Monocytes		0,80	0,35	0,60	0,26	0,471
Eos	Eosinophils		5,27	0,64	4,77	0,68	0,405
Baso	Basophils		0,00	0,00	0,03	0,06	0,374
LUC	Large Unstained Cells		0,07	0,12	0,00	0,00	0,374
LI	The lobularity index		1,77	0,04	1,74	0,07	0,545
MPXI	The myeloperoxidase index		59,37	7,77	67,57	0,84	0,143
WBCP	WBC count		0,86	0,03	0,61	0,41	0,356
%hypo	percentage of hypochromic red blood cell		0,50	0,35	0,30	0,00	0,374
%macro	Percent of red blood cells larger than120 fL		0,20	0,17	0,03	0,06	0,189
%micro	Percent of red blood cells smaller than 60 fL		0,67	0,06	0,17	0,06	0,000
RBCFragments	measurement of RBC fragments in a blood sample		0,02	0,01	0,02	0,01	1,000
RBCGhosts	the number of events in the RBC Ghost area		0,00	0,00	0,00	0,00	n/a
Neut X	the mean value of Side Scatter		9,37	0,29	9,53	0,29	0,519
Neut Y	side scatter fluorescence of the neutrophil population		16,83	0,06	16,23	0,67	0,195
MNx	mononuclear_population on the x axis.		20,33	0,29	21,23	0,87	0,165
Mny	mononuclear_population on the Y axis.		11,50	0,00	11,50	0,00	n/a
%MN	%manganese		92,33	3,06	92,43	4,50	0,976
%PMN	%Polymorphonuclear leukocytes, or PMNs		6,43	1,44	7,20	4,77	0,803
Cellular HGB	cell haemoglobin concentration		2,67	0,23	2,67	0,15	1,000

Supplementary Table 6: Peak Table for HPLC of TEM30 (Sup Fig 16)

Peak#	*Ret. Time*	Area	Height	Area %
1	10.705	1669	145	0.015
2	12.432	1549	324	0.014
3	*12.611*	1130	167	0.01
4	*12.748*	1039	228	0.01
5	13.461	6663	648	0.061
6	13.587	3777	618	0.035
7	*13.7*	7259	591	0.067
8	13.982	1318	255	0.012
9	14.425	10329	1613	0.095
10	14.931	10624610	1694039	98.046

11	*15.473*	5490	1305	0.051
12	15.614	67378	12477	0.622
13	15.842	20860	2919	0.192
14	15.984	14046	1955	0.13
15	*16.243*	23817	4305	0.22
16	16.481	7929	1621	0.073
17	16.644	1141	327	0.011
18	16.785	19938	3648	0.184
19	16.939	4375	1089	0.04
20	17.083	2530	360	0.023
21	17.231	2523	521	0.023
22	17.991	2618	209	0.024
23	20.929	1040	110	0.01
24	22.667	2055	180	0.019
25	22.79	1257	227	0.012
**Total**		10836339	1729881	100

Supplementary Table 7: Peak Table for HPLC of TEM31 (Sup Fig 16)

Peak#	Ret. Time	Area	Height	Area %
1	11.597	1482	178	0.055
2	12.777	1007	92	0.038
3	13.514	6129	534	0.228
4	13.64	5243	575	0.195
5	13.799	2850	418	0.106
6	13.935	2394	291	0.089
7	14.267	8871	1350	0.331
8	14.996	40679	4569	1.516
9	15.19	2554432	475263	95.203
10	15.596	38714	5477	1.443
11	15.784	5066	752	0.189
12	16.075	2608	483	0.097
13	16.182	6237	846	0.232
14	16.43	1656	373	0.062
15	16.636	1405	283	0.052
16	17.304	2074	392	0.077
17	23.707	1173	179	0.044
18	24.796	1130	155	0.042
Total		2683150	492212	100

Supplementary Table 8: Peak Table for HPLC of TEM32 (Sup Fig 16)

Peak#	Ret. Time	Area	Height	Area %
1	9.044	1370	139	0.031
2	10.904	1190	93	0.027
3	13.585	15536	770	0.349

4	13.811	5388	562	0.121
5	14.248	6454	1375	0.145
6	14.683	5985	663	0.135
7	14.858	42204	7296	0.949
8	14.986	4319357	788939	97.103
9	15.363	16916	2351	0.38
10	15.425	15211	1715	0.342
11	16.308	1461	168	0.033
12	16.948	13598	2371	0.306
13	17.675	1015	126	0.023
14	17.863	1506	272	0.034
15	22.788	1014	148	0.023
Total		4448205	806988	100

Supplementary Table 9: Peak Table for HPLC of TEM33 (Sup Fig 16)

Peak#	Ret. Time	Area	Height	Area%
1	8.827	5101	485	0.038
2	9.016	17645	558	0.13
3	9.942	1100	95	0.008
4	11.377	36320	3969	0.267
5	11.711	11427	1575	0.084
6	11.818	9602	1583	0.071
7	11.919	7708	1219	0.057
8	12.158	3742	360	0.028
9	12.949	13446406	1361751	98.873
10	13.448	3953	724	0.029
11	13.667	6664	1066	0.049
12	13.784	5018	946	0.037
13	14.365	1797	195	0.013
14	15.783	13185	1350	0.097
15	17.118	1540	82	0.011
16	17.8	3213	204	0.024
17	18.015	4304	455	0.032
18	18.701	3615	190	0.027
19	19.57	1854	105	0.014
20	20.521	1023	61	0.008
21	22.234	1910	125	0.014
22	22.804	2037	187	0.015
23	23.496	2491	167	0.018
24	23.859	2505	198	0.018
25	24.253	1181	108	0.009
26	24.602	4308	251	0.032
Total		13599650	1378010	100

Supplementary Notes

Supplementary Note 1

The inorganic PolyPs are found abundantly in bacterial cells and are essential for their growth and survival [5], with a concentration range of 0.1-50 mM in *E. coli* [6]. Bacteria lacking the PolyP synthesis enzyme (polyphosphate kinase 1, PPK1) show defects in motility, quorum sensing, biofilm formation and virulence [5]. Bacteria can increase intracellular PolyP concertation 1000-fold upon stress, such as nutrition limitation, oxidative stress, protein aggregation, etc [5-8]. It has even been proposed that lowering the PolyP concentration in bacteria can be an attractive antimicrobial strategy. Both new antimicrobial agents targeting PolyP synthesis enzyme PPK1 [9], as well as a clinically used drug (mesalamine) for treating patients with ulcerative colitis [10], can reduce PolyP concentration and thereby reducing bacterial invasive ability and sensitizing bacteria to oxidative inflammatory oxidants.

Supplementary References

1 Cantu, C. & Palzkill, T. The Role of Residue 238 of TEM-1 β-Lactamase in the Hydrolysis of Extended-spectrum Antibiotics. *J. Biol. Chem.* **273**, 26603-26609 (1998). https://doi.org:10.1074/jbc.273.41.26603

2 Kumar, G., Issa, B., Kar, D., Biswal, S. & Ghosh, A. S. E152A substitution drastically affects NDM-5 activity. fnx008 (2017). https://doi.org:10.1093/femsle/fnx008

3 Docquier, J.-D. *et al.* Crystal Structure of the OXA-48 β-Lactamase Reveals Mechanistic Diversity among Class D Carbapenemases. *Chem. Biol.* **16**, 540-547 (2009). https://doi.org:10.1016/j.chembiol.2009.04.010

4 Franceschini, N. *et al.* Purification and Biochemical Characterization of the VIM-1 Metallo-beta -Lactamase. **44**, 3003-3007 (2000). https://doi.org:10.1128/aac.44.11.3003-3007.2000

5 Rao, N. N., Gomez-Garcia, M. R. & Kornberg, A. Inorganic polyphosphate: essential for growth and survival. *Annu Rev Biochem* **78**, 605-647 (2009). https://doi.org:10.1146/annurev.biochem.77.083007.093039

6 Kornberg, A., Rao, N. N. & Ault-Riche, D. Inorganic polyphosphate: a molecule of many functions. *Annu Rev Biochem* **68**, 89-125 (1999). https://doi.org:10.1146/annurev.biochem.68.1.89

7 Gray, M. J. *et al.* Polyphosphate is a primordial chaperone. *Mol Cell* **53**, 689-699 (2014). https://doi.org:10.1016/j.molcel.2014.01.012

8 Reichmann, D., Voth, W. & Jakob, U. Maintaining a Healthy Proteome during Oxidative Stress. *Mol Cell* **69**, 203-213 (2018). https://doi.org:10.1016/j.molcel.2017.12.021

9 Peng, L. *et al.* Discovery and antibacterial study of potential PPK1 inhibitors against uropathogenic E. coli. *J Enzyme Inhib Med Chem* **35**, 1224-1232 (2020). https://doi.org:10.1080/14756366.2020.1766453

10 Dahl, J. U. *et al.* The anti-inflammatory drug mesalamine targets bacterial polyphosphate accumulation. *Nat Microbiol* **2**, 16267 (2017). https://doi.org:10.1038/nmicrobiol.2016.267
