# Supplementary material for: Exploiting the aggregation propensity of beta-lactamases to design inhibitors that induce enzyme misfolding
Source: Nat Commun. 2023 Sep 9;14:5571. doi: 10.1038/s41467-023-41191-z (PMC10492782; doi:10.1038/s41467-023-41191-z)
Supplement: Supplementary file 3 — Reporting Summary [file 41467_2023_41191_MOESM3_ESM.pdf]

nature portfolio

Corresponding author(s): Joost Schymkowitz and Frederic Rousseau

Last updated by author(s): Jan 21, 2023

# Reporting Summary

Nature Portfolio wishes to improve the reproducibility of the work that we publish. This form provides structure for consistency and transparency in reporting. For further information on Nature Portfolio policies, see our Editorial Policies and the Editorial Policy Checklist.

## Statistics

For all statistical analyses, confirm that the following items are present in the figure legend, table legend, main text, or Methods section.

| n/a | Confirmed | |
|---|---|---|
| ☐ | ☒ | The exact sample size (*n*) for each experimental group/condition, given as a discrete number and unit of measurement |
| ☐ | ☒ | A statement on whether measurements were taken from distinct samples or whether the same sample was measured repeatedly |
| ☐ | ☒ | The statistical test(s) used AND whether they are one- or two-sided<br>*Only common tests should be described solely by name; describe more complex techniques in the Methods section.* |
| ☒ | ☐ | A description of all covariates tested |
| ☒ | ☐ | A description of any assumptions or corrections, such as tests of normality and adjustment for multiple comparisons |
| ☐ | ☒ | A full description of the statistical parameters including central tendency (e.g. means) or other basic estimates (e.g. regression coefficient) AND variation (e.g. standard deviation) or associated estimates of uncertainty (e.g. confidence intervals) |
| ☐ | ☒ | For null hypothesis testing, the test statistic (e.g. *F*, *t*, *r*) with confidence intervals, effect sizes, degrees of freedom and *P* value noted<br>*Give P values as exact values whenever suitable.* |
| ☒ | ☐ | For Bayesian analysis, information on the choice of priors and Markov chain Monte Carlo settings |
| ☒ | ☐ | For hierarchical and complex designs, identification of the appropriate level for tests and full reporting of outcomes |
| ☒ | ☐ | Estimates of effect sizes (e.g. Cohen's *d*, Pearson's *r*), indicating how they were calculated |

*Our web collection on statistics for biologists contains articles on many of the points above.*

## Software and code

Policy information about availability of computer code

| | |
|---|---|
| Data collection | - AKTA UNICORN 7.3<br>- UNCLE Client V3.2<br>- Wyatt Dynamics 7.8.1.3<br>- FlowJo v9 |
| Data analysis | - R v4.2.2<br>- R Studio 2023.03.1+446<br>- TANGO basic academic version<br>- FoldX 3.0 Beta 6 (c)<br>- Prism 10 for MacOS |

For manuscripts utilizing custom algorithms or software that are central to the research but not yet described in published literature, software must be made available to editors and reviewers. We strongly encourage code deposition in a community repository (e.g. GitHub). See the Nature Portfolio guidelines for submitting code & software for further information.

## Data

Policy information about availability of data

All manuscripts must include a data availability statement. This statement should provide the following information, where applicable:

- Accession codes, unique identifiers, or web links for publicly available datasets
- A description of any restrictions on data availability
- For clinical datasets or third party data, please ensure that the statement adheres to our policy

The datasets generated during and/or analysed during the current study are available from the corresponding authors on reasonable request. The code of all analysis scripts and in silico datasets used are available from the corresponding authors on reasonable request.

## Human research participants

Policy information about studies involving human research participants and Sex and Gender in Research.

| | |
|---|---|
| Reporting on sex and gender | Blood samples were completely anonymized prior to transfer to our facilities. We have no information on sex and gender. |
| Population characteristics | In full compliance with the rules and regulations of our local ethical committee and the supplier (the Red Cross Flanders), we have no information on population characteristcs of blood donors used in this study. These data are to be seen as case studies only. |
| Recruitment | All blood samples were obtained from healthy volunteers from the biobank of the Red Cross Flanders in according with all relevant national legislation, including informed consent. Blood samples were completely anonymized prior to transfer to our facilities. |
| Ethics oversight | Ethical approval was obtained from the medical ethical committee of the University Hospitals Leuven for (study number S60497). |

Note that full information on the approval of the study protocol must also be provided in the manuscript.

# Field-specific reporting

Please select the one below that is the best fit for your research. If you are not sure, read the appropriate sections before making your selection.

☒ Life sciences ☐ Behavioural & social sciences ☐ Ecological, evolutionary & environmental sciences

For a reference copy of the document with all sections, see nature.com/documents/nr-reporting-summary-flat.pdf

# Life sciences study design

All studies must disclose on these points even when the disclosure is negative.

| | |
|---|---|
| Sample size | A minimum sample sizes of 3 independent experiments for all experiments was used. Often, more independent experiments were performed. This is noted in each individual figure legend. For the efficacy testing in vivo, power analysis was performed to determine sample size. Sample size calculations are typically not relevant for biophysics and biochemistry, since these methods have a very low intrinsic level of variation compared to effect size that are typically observed. In contrast replication and orthogonality are used to assess robustness and reliability. |
| Data exclusions | In the in vivo experiment, one tissue sample has been excluded (detailed in the manuscript). No other data has been excluded in this study. |
| Replication | At least three independent repeats were performed in every experiment. All attempts at replication were successful. |
| Randomization | In the mouse experiments, animals were randomly assigned to experimental groups. Randomization is not relevant for the biochemical, biophysical and cellular experiments that we performed in this study. The fact that we used the recombinantly prepared proteins and peptides and their assemblies, and studied about their properties using many biochemical, biophysical and cell biology methods in vitro with independent repeats of 3 and more times, and with the calculation of statistics from the repeats where it can be derived, randomization is not relevant for this reason. |
| Blinding | In vivo experiments were carried out in double blinded fashion. blinding is not used when employing biochemical/biophysical experiments. It is also for the reason that the researcher who conducted the experiments also carried out the analyses of the results and interpretation. However, validation of the results were confirmed by different researchers employing different standard protocols/tools |

# Reporting for specific materials, systems and methods

We require information from authors about some types of materials, experimental systems and methods used in many studies. Here, indicate whether each material, system or method listed is relevant to your study. If you are not sure if a list item applies to your research, read the appropriate section before selecting a response.

## Materials & experimental systems

| n/a | Involved in the study | |
|---|---|---|
| ☐ | ☒ | Antibodies |
| ☐ | ☒ | Eukaryotic cell lines |
| ☒ | ☐ | Palaeontology and archaeology |
| ☐ | ☒ | Animals and other organisms |
| ☒ | ☐ | Clinical data |
| ☒ | ☐ | Dual use research of concern |

## Methods

| n/a | Involved in the study | |
|---|---|---|
| ☒ | ☐ | ChIP-seq |
| ☐ | ☒ | Flow cytometry |
| ☒ | ☐ | MRI-based neuroimaging |

# Antibodies

| | |
|---|---|
| Antibodies used | - Monoclonal anti-TEM (supplier: Abcam, catalogue: ab12251, Lot number: 8A5.A10) => 1/ 5000 dilution<br>- Polyclonal rabbit anti-SHV (supplier: custom-made by Eurogentec, ZBE19010, Reference Order: 219010632 => 1/5000 dilution<br>- Chicken polyclonal anti-beta Galactosidase (supplier: Abcam, catalogue: ab106567)=> 1/10000 dilution<br>- Goat Anti-Mouse IgG HRP secondary antibodies (supplier: Abcam, catalogue: ab97040) => 1/10000 dilution<br>- Rabbit Anti-Mouse IgG HRP (supplier: Abcam , catalogue: ab6728) => 1/10000 dilution<br>- Goat Anti-Chicken HRP (supplier: Abcam , catalogue: ab97135) => 1/10000 dilution |
| Validation | All antibodies were obtained from commercial sources and have been heavily referenced. In addition, the antibodies were validated by immunoblotting by molecular weight. |

# Eukaryotic cell lines

Policy information about cell lines and Sex and Gender in Research

| | |
|---|---|
| Cell line source(s) | HEK-293 ATCC (CRL-1573)<br>SH-SY5Y ATCC (CRL-2266)<br>Human Umbilical Vein Endothelial Cells, Lonza (HUVEC pooled donor S-part EGM-2, Ref C2519AS AMP)<br>Primary Renal Proximal Tubule Epithelial Cells; Normal, Human (RPTEC) (ATCC® PCS-400-010™) |
| Authentication | Cell lines were verifed by Eurofins Genomics Europe (accredited acc.to DIN EN ISO/IEC 17025:200). Genetic characteristics were determined by PCR-single-locus-technology. 16 independent PCR-systems D8S1179, D21S11, D7S820, CSF1PO, D3S1358, TH01, D13S317, D16S539, D2S1338, AMEL, D5S818, FGA, D19S433, vWA, TPOX and D18S51 were investigated. Results were compared with the online database of the DSMZ (http://www.dsmz.de/de/service/services-human-and-animal-cell) and the Cellosaurus database (https://web.expasy.org/cellosaurus. Only the PCR-systems with ANSI/ATCC standard ASN-0002 were aligned in the final comparison. |
| Mycoplasma contamination | A Mycoplasma detection kit (Cat# M7006) was used to confirm the absence of Mycoplasma infections. |
| Commonly misidentified lines<br>(See ICLAC register) | we have not used commonly misidentified cell lines |

# Animals and other research organisms

Policy information about studies involving animals; ARRIVE guidelines recommended for reporting animal research, and Sex and Gender in Research

| | |
|---|---|
| Laboratory animals | Female C57BL/6Jax mice of 6 to 8 weeks with uniform weight (20 and 23g) were used in this study (Harlan, The Netherlands). Mice house in Individually Ventilated Cages (IVC). These cages are all individually ventilated which receive HEPA filtered air from a blower and have a separate air in- and outlet per cage to minimize the risks for transmission of pathogens, especially airborne pathogens, between cages, and significantly reduce the number of allergens. The bedding for mice is composed of spruce particles of approximately 2.5 - 3.5 mm, type Lignocel® BK 8/15. Temperature is checked daily 22+2C °. Humidity is between 45-60%. |
| Wild animals | No wild animals were used in this study. |
| Reporting on sex | Female mice were used for the bladder infection model. |
| Field-collected samples | No field collected samples were used in this study. |
| Ethics oversight | All mouse experiments were conducted according to the national (Belgian Law 14/08/1986 and 22/12/2003, Belgian Royal Decree 06/04/2010) and European (EU Directives 2010/63/EU, 86/609/EEG) animal regulations. All protocols were approved by the Institutional ethics committee on animal experimentation of the KU Leuven. All relevant animal characteristics and housing conditions are specified in the materials and methods. |

Note that full information on the approval of the study protocol must also be provided in the manuscript.

# Flow Cytometry

## Plots

Confirm that:

☒ The axis labels state the marker and fluorochrome used (e.g. CD4-FITC).

☒ The axis scales are clearly visible. Include numbers along axes only for bottom left plot of group (a 'group' is an analysis of identical markers).

☒ All plots are contour plots with outliers or pseudocolor plots.

☒ A numerical value for number of cells or percentage (with statistics) is provided.

## Methodology

| | |
|---|---|
| Sample preparation | Bacterial cell cultures were prepared as standard, using treatments as indicated in the manuscript. |
| Instrument | GalliosTM Flow Cytometer (Beckman  Coulter, USA) |
| Software | Flowjo software version 10.6. (BD Biosciences) |
| Cell population abundance | At least 40.000 cells were analysed per condition |
| Gating strategy | Cells populations were analysed only, not sorted. |

☐ Tick this box to confirm that a figure exemplifying the gating strategy is provided in the Supplementary Information.
